# Deep superpixel generation and clustering for weakly supervised segmentation of brain tumors in MR images

**DOI:** 10.1186/s12880-024-01523-x

**Published:** 2024-12-18

**Authors:** Jay J. Yoo, Khashayar Namdar, Farzad Khalvati

**Affiliations:** 1 Institute of Medical Science, 1 King’s College Circle, Toronto, M5S 1A8 Ontario Canada; 2 Department of Diagnostic & Interventional Radiology, The Hospital for Sick Children, 555 University Avenue, Toronto, M5G 1X8 Ontario Canada; 3 Department of Medical Imaging, University of Toronto, 263 McCaul Street, Toronto, M5T 1W7 Ontario Canada; 4 Department of Computer Science, University of Toronto, 40 St. George Street, Toronto, M5S 2E4 Ontario Canada; 5 Department of Mechanical and Industrial Engineering, University of Toronto, 5 King’s College Road, Toronto, M5S 3G8 Ontario Canada; 6 Vector Institute, 661 University Avenue, Toronto, M5G 1M1 Ontario Canada

**Keywords:** Image segmentation, Weakly supervised learning, Convolutional neural networks, Superpixels, Glioma, Magnetic resonance imaging

## Abstract

**Purpose:**

Training machine learning models to segment tumors and other anomalies in medical images is an important step for developing diagnostic tools but generally requires manually annotated ground truth segmentations, which necessitates significant time and resources. We aim to develop a pipeline that can be trained using readily accessible binary image-level classification labels, to effectively segment regions of interest without requiring ground truth annotations.

**Methods:**

This work proposes the use of a deep superpixel generation model and a deep superpixel clustering model trained simultaneously to output weakly supervised brain tumor segmentations. The superpixel generation model’s output is selected and clustered together by the superpixel clustering model. Additionally, we train a classifier using binary image-level labels (i.e., labels indicating whether an image contains a tumor), which is used to guide the training by localizing undersegmented seeds as a loss term. The proposed simultaneous use of superpixel generation and clustering models, and the guided localization approach allow for the output weakly supervised tumor segmentations to capture contextual information that is propagated to both models during training, resulting in superpixels that specifically contour the tumors. We evaluate the performance of the pipeline using Dice coefficient and 95% Hausdorff distance (HD95) and compare the performance to state-of-the-art baselines. These baselines include the state-of-the-art weakly supervised segmentation method using both seeds and superpixels (CAM-S), and the Segment Anything Model (SAM).

**Results:**

We used 2D slices of magnetic resonance brain scans from the Multimodal Brain Tumor Segmentation Challenge (BraTS) 2020 dataset and labels indicating the presence of tumors to train and evaluate the pipeline. On an external test cohort from the BraTS 2023 dataset, our method achieved a mean Dice coefficient of 0.745 and a mean HD95 of 20.8, outperforming all baselines, including CAM-S and SAM, which resulted in mean Dice coefficients of 0.646 and 0.641, and mean HD95 of 21.2 and 27.3, respectively.

**Conclusion:**

The proposed combination of deep superpixel generation, deep superpixel clustering, and the incorporation of undersegmented seeds as a loss term improves weakly supervised segmentation.

## Introduction

Segmentation is crucial in medical imaging for localizing regions of interest (ROI), which can then assist in the identification of anomalies. Machine learning (ML) can automate the analysis and segmentation of medical images with excellent performance [1], on computed tomography [2], ultrasound [3, 4], and magnetic resonance (MR images) [5–7]. However, training ML segmentation models demands large datasets of manually annotated medical images which are not only tedious and expensive to acquire, but also may be inaccessible for specific diseases such as rare cancers.

In brain tumor analysis, ML segmentation models and supervised learning have demonstrated the ability to annotate ROIs in MR images when a sufficient number of manually annotated patient scans are available [8]. For specific tumor types, such as pediatric low-grade gliomas, accurate ROI segmentation is required for downstream tasks, including molecular subtype identification and treatment planning [9]. However, conventional supervised learning approaches are limited by their dependence on large annotated datasets, making them impractical in scenarios with insufficient labeled data.

Alternative frameworks, including unsupervised learning, transfer learning, multitask learning, semi-supervised learning, and weakly supervised learning, have been developed to address these limitations. Unsupervised learning, while applicable in the absence of labeled data, typically yields lower performance [10]. Transfer learning leverages patterns learned from external datasets, enabling improved performance with limited annotated data [11]. Multitask learning facilitates simultaneous training on primary and auxiliary tasks when multiple ground truth labels are available [12]. Semi-supervised learning is applicable when a subset of the dataset is annotated [13], and weakly supervised learning enables segmentation models to localize anomalies using image-level classification labels [14], which are less expensive to acquire than pixel-level annotations.

This study focuses on leveraging image-level binary labels to differentiate cancerous from non-cancerous images for pixel-wise tumor segmentation, utilizing weakly supervised learning as the primary framework. The main limitation of the existing weakly supervised learning methods is the gap between them and the supervised methods in terms of performance.

Class activation maps (CAM) and attention maps are frequently used for weakly supervised tumor segmentation when the only available ground truths are image-level classification labels. The classification labels are used to train a classifier which is then used to acquire the CAMs or attention maps. CAMs have been used in a variety of medical imaging problems including the segmentation of organs [15], pulmonary nodules [16], and brain lesions [17]. Classifier architectures such as PatchConvNet have been specifically designed to generate accurate attention maps [18]. Multiple Instance Learning (MIL) is another approach to weakly supervised segmentation that trains a model using instances arranged in sets, which in this case are patches of an image, and then outputs a prediction for the whole set by aggregating the predictions corresponding to the instances within the set [19]. Additionally, a multi-level classification network (MLCN) which was designed for multi-class brain tumor segmentation has also been demonstrated to be effective for single-class segmentation [20].

Another approach to weakly supervised segmentation is to utilize superpixels. Superpixels are pixels grouped based on various characteristics, including pixel gray levels and proximity. By grouping pixels together, superpixels capture redundancy and reduce the complexity of computer vision tasks, making them valuable for image segmentation [21–23]. The Superpixel Pooling Network (SPN) is an example of a weakly supervised segmentation method that uses superpixels generated from algorithms such as Felzenszwalb’s algorithm to aid the segmentation task [23]. Superpixels generated using Simple Linear Iterative Clustering (SLIC) have also been used to refine CAMs over multiple steps to generate pseudo labels which can then be used to train a segmentation model [24]. Superpixels can also be generated using ML-based approaches such as Fully Convolutional Networks (FCN) which generate oversegmented superpixels with less computational complexity [25].

Transformers have become a prominent approach to many computer vision tasks including image segmentation. However, transformer architectures require large datasets to be effectively trained [26], which are not available for many medical contexts such as pediatric cancer. Transformers have been trained using large, varied datasets to produce foundational segmentation models that have strong zero-shot and few-shot generalization, the most notable of which is the Segment Anything Model (SAM) [27]. Foundational segmentation models have the potential to circumvent dataset requirements for medical contexts due to their ability to generalize beyond data observed during training. A foundational segmentation model for the medical space known as MedSAM has also been proposed [28], but these segmentation models are limited by a reliance on user prompts to segment specific objects. SAM requires manually selected points indicating the presence and/or absence of desired objects or manually selected bounding boxes while MedSAM specifically requires manually selected bounding boxes. Therefore, using such foundational models introduces more manual effort than image-level classification labels, unless the prompt acquisition is automated. In addition, both SAM and MedSAM require RGB image inputs, which prevent multimodal medical image inputs.

We hypothesize that superpixels can be leveraged to acquire additional contextual information, thereby improving weakly supervised segmentation performance. We propose to simultaneously train a superpixel generation model and a superpixel clustering model using localization seeds acquired from a classifier trained with the image-level labels. For each pixel, the superpixel generator assigns association scores to each potential superpixel, and the clustering model predicts weights for each superpixel based on their overlap with the tumor. Pixels are soft clustered based on their association with highly weighted superpixels to form segmentations. The superpixel models combine information from the pixel intensities of the superpixels with information from the localization seeds, yielding segmentations that are consistent with both the classifier understanding from the localization seeds and the pixel intensities of the MR images.

The novelty of the work is summarized by the following points:

- We combine a deep superpixel generation and clustering module into a weakly supervised brain tumor segmentation pipeline that improves performance of the models on MR images.
- We derive image-specific masks, which are referred to as localization seeds, from binary classifier trained to identify cancerous images. We propose the use of undersegmented seeds rather than more accurate seeds as priors for training the models, and demonstrate that using the undersegmented seeds leads to improved weakly supervised segmentation.
- We compare our proposed algorithm with multiple benchmark models such as foundational models, supervised learning, and other superpixel-based weakly supervised methods.

To outline the structure of this manuscript beyond the introduction, the Materials and methods section presents the datasets, the data preprocessing, the proposed methodology, the implementation details, and the metrics used for evaluation. The Results section presents the performance of the proposed pipeline, baseline methods, and variants of the proposed pipeline, including ablation studies. The Discussion section discusses the key findings and limitations of the research, and the Conclusions section presents the conclusions of the work.

## Materials and methods

### Datasets and preprocessing

Similar to other state-of-the-art brain tumor segmentation models [8, 29], the proposed pipeline relies on multimodal 4-channel MR images as inputs. As such, we form our dataset using the 369 3-dimensional (3D) T1-weighted, post-contrast T1-weighted, T2-weighted, and T2 Fluid Attenuated Inversion Recovery (T2-FLAIR) MR image volumes from the Multimodal Brain Tumor Segmentation Challenge (BraTS) 2020 dataset [30–34]. These volumes were combined to form 369 3D multimodal volumes with 4 channels, where the channels represent the T1-weighted, post-contrast T1-weighted, T2-weighted, and T2-FLAIR images for each patient. Only the training set of the BraTS dataset was used because it is the only one with publicly available ground truths.

The images were preprocessed by first cropping each image and segmentation map using the smallest bounding box which contained the brain, clipping all non-zero intensity values to their 1 and 99 percentiles to remove outliers, normalizing the cropped images using min-max scaling, and then randomly cropping the images to fixed patches of size $128 \times 128$ along the coronal and sagittal axes, as done by Henry et al. [5] and Wang et al. [35] in their work with BraTS datasets. The 369 available patient volumes were then split into 295 (80%), 37 (10%), and 37 (10%) volumes for the training, validation, and test cohorts, respectively.

The 3D multimodal volumes were then split into axial slices to form multimodal 2-dimensional (2D) images with 4 channels. After splitting the volumes into 2D images, the first 30 and last 30 slices of each volume were removed, as done by Han et al. [36] because these slices lack useful information. The training, validation, and test cohorts had 24635, 3095, and 3077 stacked 2D images, respectively. For the training, validation, and test cohorts, respectively; 68.9%, 66.3%, and 72.3% of images were cancerous. The images will be referred to as $X = \{x_1, x_2, ..., x_N\} \in \mathbb {R}^{N, 4, H, W}$, where N is the number of images, $H=128$, and $W=128$. Ground truths for each slice $y_k$ were assigned 0 if the corresponding true segmentations were empty, and 1 otherwise.

To assess generalizability, we also prepared the BraTS 2023 dataset [30–32, 34, 37] for use as an external test cohort during evaluation. To do so, we removed data from the BraTS 2023 dataset that appeared in the BraTS 2020 dataset, preprocessed the images as was done for the images in the BraTS 2020 dataset, and then extracted the cross-section with the largest tumor area from each patient. This resulted in 886 images from the BraTS 2023 dataset.

### Proposed weakly supervised segmentation method

We first trained a classifier model to identify whether an image contains a tumor, then generated localization seeds from the model using Randomized Input Sampling for Explanation of Black-box Models (RISE) [38]. The localization seeds used the classifier’s understanding to assign each pixel in the images to one of three categories. The first, referred to as positive seeds, indicate regions of the image with a high likelihood of containing a tumor. The second, referred to as negative seeds, indicate regions with a low likelihood of containing a tumor. The final category, referred to as unseeded regions, correspond to the remaining areas of the images and indicated regions of low confidence from the classifier. This resulted in positive seeds that undersegment the tumor, and negative seeds that undersegment the non-cancerous regions. Assuming that the seeds were accurate, these seeds simplified the task of classifying all the pixels in the image to classifying all the unseeded regions in the image, and provided a prior on image features indicating the presence of tumors. The seeds were used as pseudo-ground truths to simultaneously train both a superpixel generator and a superpixel clustering model which, when used together, produced the final refined segmentations from the probability heat map of the superpixel-based segmentations. Using undersegmented seeds, rather than seeds that attempt to precisely replicate the ground truths, increased the acceptable margin of error and reduced the risk of accumulated propagation errors.

**Fig. 1:**
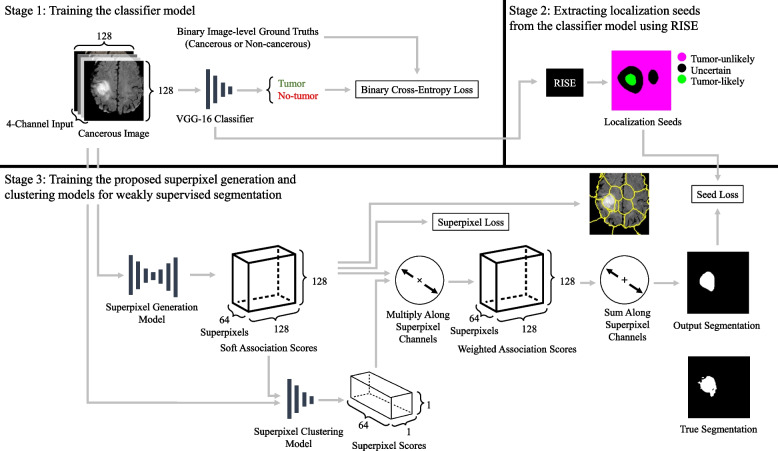
Flowchart of proposed weakly supervised segmentation method. For the localization seeds component; green indicates positive seeds, magenta indicates negative seeds, black indicates unseeded regions. Solid lines represent use as inputs and outputs. The superpixel generation model uses a fully convolutional AINet architecture [40] and outputs each pixel’s association with each of the 64 potential superpixels. The superpixel clustering network uses a ResNet-18 architecture and outputs a score for each of the 64 superpixels indicating the likelihood that each superpixel contains a tumor. The labels used to train the method are binary image-level labels which indicate the presence or absence of tumors

A flowchart of the proposed methodology is presented in Fig. 1. We chose to use 2D images over 3D images because converting 3D MR volumes to 2D MR images yields significantly more data samples and reduces memory costs. Many state-of-the-art models such as SAM and MedSAM use 2D images [27, 28], and previous work demonstrated that brain tumors can be effectively segmented from 2D images [39].

#### Stage 1: Training the classifier model

The classifier model was trained to output the probability that each $x_k \in X$ contains a tumor, where $X = \{x_1, x_2, ..., x_N\} \in \mathbb {R}^{N, 4, H, W}$ is a set of brain MR images, and N is the number of images in *X*. Prior to being input to the classifier, the images were upsampled by a factor of 2. The images were not upsampled for any other model in the proposed method. This classifier model was trained using $Y = \{y_1, ..., y_N\}$ as the ground truths, where $y_k$ is a binary label with a value of 1 if $x_k$ contains tumor and 0 otherwise. The methodology is independent of the classifier architecture, and thus, other classifier architectures can be used instead.

#### Stage 2: Extracting localization seeds from the classifier model using RISE

RISE is a method proposed by Petsiuk et al. that generates heat maps indicating the importance of each pixel in an input image for a given model’s prediction [38]. RISE first creates numerous random binary masks which are used to perturb the input image. RISE then evaluates the change in the model prediction when the input image is perturbed by each of the masks. The change in model prediction at each perturbed pixel is then accumulated across all the masks to form the heat maps.

We applied RISE to our classifier to generate heat maps $H_{rise} \in \mathbb {R}^{N, H, W}$ for each of the images. The heat maps indicate the approximate likelihood for tumors to be present at each pixel. These heat maps were converted to localization seeds by setting the pixels corresponding to the top 20% of values in $H_{rise}$ as positive seeds, and setting the pixels corresponding to the bottom 20% of values as negative seeds. $S_+ = \{s_{+_1}, s_{+_2}, ..., s_{+_{N}}\} \in \mathbb {R}^{N, H, W}$ is defined as a binary map indicating positive seeds and $S_- = \{s_{-_1}, s_{-_2}, ..., s_{-_{N}}\} \in \mathbb {R}^{N, H, W}$ is defined as a binary map indicating negative seeds. Any pixel not set as either a positive or negative seed was considered uncertain. Once all the seeds were generated, any images considered healthy by the classifier had their seeds replaced by new seeds. These new seeds did not include any positive seeds and instead set all pixels as negative seeds, which minimized the risk of inaccurate positive seeds from healthy images causing propagation errors.

#### Stage 3: Training the proposed superpixel generation and clustering models for weakly supervised segmentation

The superpixel generation model and the superpixel clustering model were trained to output the final segmentations without using the ground truth segmentations. The superpixel generation model assigns $N_S$ soft association scores to each pixel, where $N_S$ is the maximum number of superpixels to generate, which we set to 64. The association maps are represented by $Q = \{q_1, ..., q_{N}\} \in \mathbb {R}^{N, N_S, H, W}$, where N is the number of images in *X*, and $q_{k, s, p_y, p_x}$ is the probability that the pixel at $(p_y, p_x)$ is assigned to the superpixel *s*. Soft associations may result in a pixel having similar associations to multiple superpixels. The superpixel clustering model then assigns superpixel scores to each superpixel indicating the likelihood that each superpixel represents a cancerous region. The superpixel scores are represented by $R = \{r_1, ..., r_{N}\} \in \mathbb {R}^{N, N_S}$ where $r_{k, s}$ represents the probability that superpixel *s* contains a tumor. The pixels can then be soft clustered into a tumor segmentation by performing a weighted sum along the superpixel association scores using the superpixel scores as weights. The result of the weighted sum is the likelihood that each pixel belongs to a tumor segmentation based on its association with strongly weighted superpixels.

The superpixel generator takes input $x_k$ and outputs a corresponding value $q_k$ by passing the direct output of the superpixel generation model through a SoftMax function to rescale the outputs from 0 to 1 along the $N_s$ superpixel associations. The clustering model receives a concatenation of $x_k$ and $q_k$ as input, and the outputs of the clustering model are passed through a SoftMax function to yield superpixel scores *R*. Heatmaps $H_{spixel_+} \in \mathbb {R}^{N, H, W}$ that localize the tumors can be acquired from *Q* and *R* by multiplying each of the $N_S$ association maps in *Q* by their corresponding scores *R*, and then summing along the $N_S$ channels as shown in (1). The superpixel generator architecture is based on AINet proposed by Wang et al. [40], which is a FCN-based superpixel segmentation model that uses a variational autoencoder. The innovation introduced by AINet is the association implantation module which improves superpixel segmentation performance by allowing the model to directly perceive the associations between pixels and their surrounding candidate superpixels. We altered AINet, which outputs local superpixel associations, to output global associations instead so that *Q* could be passed into the superpixel clustering model. This allowed the generator model to be trained in tandem with the clustering model. Two different loss functions were used to train the superpixel generation and clustering models. The first loss function, $L_{spixel_+}$, was proposed by Yang et al. [25] and minimizes the variation in pixel intensities and pixel positions in each superpixel. This loss is defined in (2), where *p* represents a pixel’s coordinates ranging from (1, 1) to (*H*, *W*), and *m* is a coefficient used to tune the size of the superpixels, which we set as $\frac{3}{160}$. We selected this value for *m* by multiplying the value suggested by the original work, $\frac{3}{16000}$ [25], by 100 to achieve the desired superpixel size. $l_s$ and $u_s$ are the vectors representing the mean superpixel location and the mean superpixel intensity for superpixel *s*, respectively. The second loss function, $L_{seed}$, is a loss from the Seed, Expand, and Constrain paradigm for weakly supervised segmentation. This loss was designed to train models to output segmentations that include positive seeded regions and exclude negative seeded regions [41]. This loss is defined in (1)-(4) where C indicates whether the positive or negative seeds of an image $s_k$ is being evaluated. These losses, when combined together, encourage the models to account for both the localization seeds *S* and the pixel intensities. This results in $H_{spixel_+}$ localizing the unseeded regions that correspond to the pixel intensities in the positive seeds. The combined loss is presented in (5), where $\alpha$ is a weight for the seed loss. The output $H_{spixel_+}$ can then be thresholded to generate final segmentations $E_{spixel_+} \in \mathbb {R}^{N, H, W}$.

While the superpixel generation and clustering models were trained using all images in *X*, during inference the images predicted to be healthy by the classifier were assigned empty output segmentations.

1 $$\begin{aligned} H_{{spixel_+}_k} = \sum _{s \in N_s} Q_{k,s} R_{k,s} \end{aligned}$$

2 $$\begin{aligned} L_{spixel} = \frac{1}{N} \sum _{k=1}^{N} \sum _p \left( \left\| \sum _{s \in N_s} u_s Q_{k,s}(p) \right\| _2 + m \left\| \sum _{s \in N_s} l_s Q_{k,s}(p) \right\| _2 \right) \end{aligned}$$

3 $$\begin{aligned} H_{{spixel_-}_k} = 1 - H_{{spixel_+}_k} \end{aligned}$$

4 $$\begin{aligned} L_{seed} = \frac{1}{N} \sum _{k=0}^{N - 1} \frac{-\sum _{C \in [+, -]} \sum _{i, j \in s_{C_k}} \log {H_{{spixel_C}_k}}_{i,j}}{\sum _{C \in [+, -]} \left| {s_{C_k}}\right| } \end{aligned}$$

5 $$\begin{aligned} L = L_{spixel} + \alpha L_{seed} \end{aligned}$$

### Implementation details

For the classifier model, we used a VGG-16 architecture [42] with batch normalization, whose output was passed through a Sigmoid function. The classifier was trained to optimize the binary cross-entropy between the output probabilities and the binary ground truths using an Adam optimizer with $\beta _1 = 0.9, \beta _2 = 0.999, \epsilon =1e-8$, and a weight decay of 0.1 [43]. The classifier was trained for 100 epochs using a batch size of 32. The learning rate was initially set to $5e-4$ and then decreased by a factor of 10 when the validation loss did not decrease by $1e-4$.

When using RISE, we set the number of masks for an image to 4000 and used the same masks across all images.

For the clustering model, we used a ResNet-18 architecture [44] with batch normalization. The superpixel generation and clustering models were trained using an Adam optimizer with $\beta _1 = 0.9, \beta _2 = 0.999, \epsilon =1e-8$, a weight decay of 0.1. The models were trained for 100 epochs using a batch size of 32. The learning rate was initially set to $5e-4$, which was halved every 25 epochs. The weight for the seed loss, $\alpha$, was set to 50.

### Evaluation metrics

We evaluated the segmentations generated by our proposed weakly supervised segmentation method and comparative methods using Dice coefficient (Dice) and 95% Hausdorff distance (HD95). We also evaluated the seeds generated using RISE and seeds generated for other comparative methods using Dice, HD95, and a metric that we refer to as undersegmented Dice coefficient (U-Dice).

Dice is a common metric in image segmentation that measures the similarity between two binary segmentations. Dice compares the pixel-wise agreement between the generated and ground truth segmentations using a value from 0 to 1. 0 indicates no overlap between the two segmentations while 1 indicates perfect overlap. A smoothing factor of 1 was used to account for division by zero with empty segmentations and empty ground truths.

The Hausdorff distance is the maximum distance among all the distances from each point on the border of the generated segmentation to their closest point on the boundary of the ground truth segmentations. Therefore, Hausdorff distance represents the maximum distance between two segmentations. However, Hausdorff distance is extremely sensitive to outliers. To mitigate this limitation of the metric, we used HD95 which is the 95th percentile of the ordered distances. HD95 values of 0 indicate perfect segmentations while greater HD95 values indicate segmentations with increasingly flawed boundaries. HD95 was set to 0 when either the segmentations/seeds or the ground truths had empty segmentations.

U-Dice is an alteration to Dice that measures how much of the seeds undersegment the ground truths. We used this measure because our method assumes that the seeds undersegment the ground truths rather than precisely contouring them. Therefore, this measure can be used to determine the impact of using undersegmented seeds as opposed to more oversegmented seeds. A value of 1 indicates that the seeds perfectly undersegment the ground truths and a value of 0 indicates that the seed does not have any overlap with the ground truth. A smoothing factor of 1 was also used for the U-Dice. The equation for Dice is presented in Eq. 6 and the equation for U-Dice is presented in Eq. 7, where A is the seed or proposed segmentation and B is the ground truth.

6 $$\begin{aligned} \text {Dice} = \frac{2 | A \cap B | + 1}{| A | + | B | + 1} \end{aligned}$$

7 $$\begin{aligned} \text {U-Dice} = \left\{ \begin{array}{ll} 0 & \text {if}\ | A | = 0, | B |> 0\\ \frac{| A \cap B | + 1}{| A | + 1} & \text {otherwise} \end{array}\right. \end{aligned}$$

## Results

We trained our models using images *X* and binary image-level labels *Y* without using any segmentation ground truths. The classifier achieved a test accuracy of 0.933 using a decision threshold of 0.5. Table 1 presents the per-image Dice and HD95 between the output segmentations for our proposed method and the ground truth segmentations, with the proposed method written in bold text. The table also includes the Dice and HD95 across correctly classified images and across incorrectly classified images.

**Table 1:** Comparison of proposed weakly supervised segmentation method and baseline methods

Method	All Images				Correctly Classified Images				Incorrectly Classified Images			
	Dice		HD95		Dice		HD95		Dice		HD95	
	Test	BraTS 2023	Test	BraTS 2023	Test	BraTS 2023	Test	BraTS 2023	Test	BraTS 2023	Test	BraTS 2023
**Proposed** $\varvec{(\alpha = 50)}$	0.691	0.745	18.1	20.8	0.737	0.754	19.3	21.0	0.059	0.002	1.24	0.000
Proposed ($\alpha$ = 10)	0.594	0.574	24.9	34.7	0.622	0.580	26.7	35.1	0.206	0.002	0.549	0.000
Ablation (Seeds)	0.671	0.697	18.6	22.2	0.712	0.705	19.9	22.5	0.110	0.002	0.748	0.000
Ablation (Superpixels)	0.401	0.302	5.52	6.71	0.419	0.306	5.89	6.79	0.156	0.002	0.387	0.000
Proposed (CAM-S Seeds)	0.612	0.583	37.7	51.9	0.639	0.590	40.3	52.5	0.228	0.002	1.32	0.000
Proposed (Fully Supervised)	0.835	0.891	7.31	8.61	0.875	0.902	7.80	8.71	0.284	0.002	0.662	0.000
PatchConvNet	0.134	0.001	54.13	87.3	0.053	0.001	71.7	76.8	0.370	0.002	3.12	51.8
SAM	0.616	0.641	24.1	27.3	0.613	0.641	24.5	27.2	0.660	0.558	18.8	35.2
MLCN	0.607	0.583	28.8	51.9	0.649	0.590	30.7	52.5	0.025	0.002	2.53	0.000
SPN	0.375	0.260	53.9	73.4	0.401	0.263	57.5	74.2	0.012	0.002	4.25	0.000
MIL	0.391	0.126	47.6	53.4	0.413	0.128	50.9	54.0	0.086	0.002	2.83	0.000
CAM-S	0.658	0.646	16.7	21.2	0.693	0.653	17.9	21.5	0.168	0.002	0.728	0.000

Dice coefficients and 95% Hausdorff distances between generated segmentations and true segmentations on all images, correctly classified images, and incorrectly classified images. 24540 images in the training cohort, 2822 images in the validation cohort, 2870 images in the test cohort, and 876 images in the BraTS 2023 test cohort were correctly classified. 95 images in the training cohort, 273 images in the validation cohort, 207 images in the test cohort, and 10 images in the BraTS 2023 test cohort were incorrectly classified

We also present the performance of baseline methods for comparison. The first baseline method is the proposed method using a seed loss weight of 10 ($\alpha = 10$) rather than a seed loss weight of 50 ($\alpha = 50$). This is to determine the impact of the seed loss weight on the segmentation performance. The second baseline method is the performance of the AINet architecture used by the superpixel generator model with the superpixel components removed and altered to directly output segmentations. This method, referred to as Ablation (Seeds), serves as an ablation study that investigates the impact of removing the superpixel generation component from the proposed method. The third baseline method is the performance of the superpixel model with the superpixel clustering component removed. For this baseline, instead of using the superpixel clustering model to select superpixels, a superpixel was selected if a majority of the superpixel overlapped with positive seeds from RISE. This method is referred to as Ablation (Superpixels), and presents the impact of removing the superpixel clustering component from the proposed method. The fourth baseline method is our proposed method with the VGG-16 classifier replaced by a PatchConvNet classifier [18]. For this baseline, we used the attention maps from PatchConvNet in place of the RISE generated seeds to train our superpixel generation and clustering models. The fifth baseline method is a pretrained SAM model provided by Meta [27]. As SAM was trained on RGB images, we used the T2-FLAIR channel of each image converted to RGB as inputs to the SAM baseline. To generate the user prompts required by SAM to segment specific regions, for each image we used the center of mass for the largest positive seed region from RISE as a positive object point and the center of masses of all negative seed regions from RISE as negative object points. The sixth baseline is the MLCN method simplified to be applicable for single-class segmentation as described in the original work [20]. For the seventh baseline, we evaluated the performance of our proposed method when trained in a fully supervised fashion rather than a weakly supervised fashion to evaluate the performance gap between weakly supervised and fully supervised segmentation.

In addition, we compared our proposed method with three other methods designed for weakly supervised segmentation. The first is the SPN which relies on pre-generated superpixels [23], which we generated using Felzenszwalb’s Algorithm with a scale of 100 and a standard deviation of 0.5. resulting in approximately 100 pre-generated superpixels per image. The second is a MIL baseline that we trained using a VGG-16 model with batch normalization [19]. At each epoch, we extracted 50 patches of shape $128 \times 128$ from the images after upsampling them to $512 \times 512$. At each iteration, we set the 20% of patches with the highest predictions to be cancerous and 20% of patches with the lowest predictions to be non-cancerous, as these thresholds were demonstrated to be effective in the original work and are consistent with the thresholds we used when generating seeds using RISE. The third is a weakly supervised segmentation method for cardiac adipose tissue which we will refer to as the CAM-Superpixels (CAM-S) method [24]. This method generates CAMs from a classifier and then uses pre-generated superpixels to refine the CAMs into pseudo-labels over multiple steps, which are then used to train a segmentation model. In addition, we also evaluated the performance of our model when trained with the pseudo labels generated from the CAM-S method instead of the localization seeds generated using RISE.

The SPN, MLCN, SPN, MIL, and CAM-S methods differ from the other baselines in that they are not variants of the proposed method, and thus do not assign empty segmentations to images classified as non-cancerous. To allow for effective comparison, we present the performance of these baseline methods with empty segmentations assigned to images classified as non-cancerous by the classifier trained for our proposed method.

In addition, we present the generalizability of each model by evaluating the models on the BraTS 2023 test cohort. These results can be found under the BraTS 2023 columns in Table 1.

Each of the presented methods uses a decision threshold to convert the output probability maps to binary segmentations. The decision threshold for each method was determined by evaluating the Dice on the validation cohort at threshold intervals of 0.1 and choosing the threshold that yielded the maximum validation Dice. The proposed methods used thresholds of 0.6 and 0.9 for seed loss weights of 50 and 10, respectively, while the ablation (seeds) and PatchConvNet methods used thresholds of 0.5 and 0.9, respectively. The ablation (superpixels) method did not use a decision threshold and instead set superpixels with more than 50% overlap with positive seeds as segmented regions. The proposed method trained using CAM-S seeds used a threshold of 0.9 and the proposed method trained under fully supervised training used a threshold of 0.8. The SPN, MIL, MLCN, and CAM-S models used thresholds of 0.9, 0.3, 0.1, and 0.8 respectively.

**Fig. 2:**
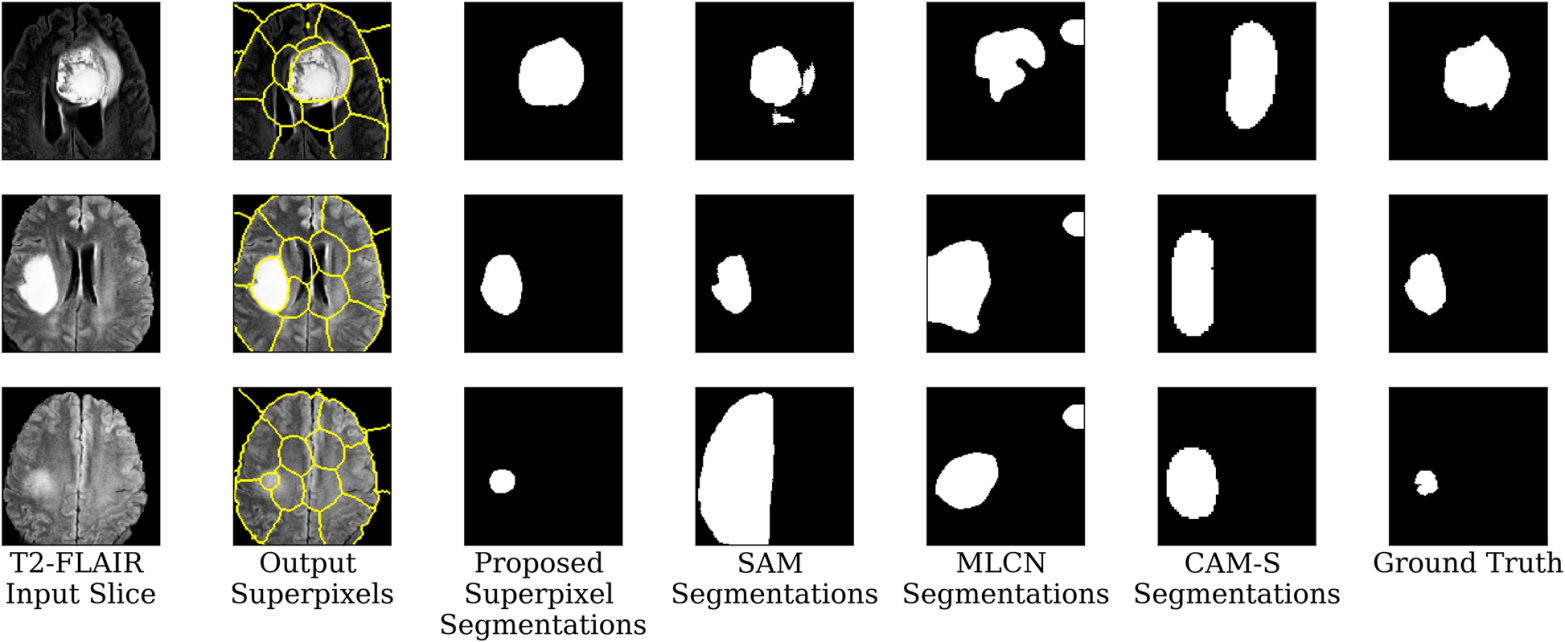
Visualization of T2-FLAIR channel of MR images, generated superpixels, output segmentations from the proposed method, output segmentations from the SAM, MLCN, and CAM-S baselines, and the true segmentations for three examples

Figure 2 presents three images from the test set and their corresponding segmentations generated at each step of the pipeline. In addition, Fig. 2 also presents the outputs from the SAM, MLCN, and CAM-S baselines, as well as the true segmentations. The SAM, MLCN, and CAM-S baselines are the only visualized baselines because they were the only baseline methods with comparable performance to the proposed method.

Table 2 presents the Dice, HD95 and U-Dice of the RISE seeds used for the proposed method and the other seeds used in the comparative methods. These results serve to indicate the potential propagation errors from the seeds and explain the performance change when training the proposed method using different seeds as presented in Table 1. It can be seen that the CAM-S seeds outperformed the positive seeds generated using RISE in terms of both Dice and HD95 but was worse than the positive seeds in terms of U-Dice, indicating that the CAM-S seeds were more accurate to the tumors but the positive seeds from RISE better undersegmented the tumors. Considering that in Table 1, the proposed method achieved Dice of 0.745 on the BraTS 2023 test cohort, while the proposed method trained on the CAM-S and PatchConvNet seeds achieved Dice of 0.583 and 0.134, respectively, this demonstrates that using undersegmented seeds instead of more accurate seeds can lead to improved performance.

**Table 2:** Comparison of seeds generated using RISE, PatchConvNet and CAM-S

Seeds	Dice		HD95		U-Dice	
	Test	BraTS 2023	Test	BraTS 2023	Test	BraTS 2023
RISE (Positive)	0.485	0.522	26.0	25.3	0.732	0.900
RISE (Negative)	0.946	0.922	0.000	0.000	0.900	0.857
PatchConvNet	0.005	0.001	69.3	89.9	0.026	0.015
CAM-S	0.610	0.594	19.3	27.2	0.560	0.569

Dice coefficients, 95% Hausdorff distances, and undersegmented Dice coefficients between the seeds generated for the evaluated methods and true segmentations

## Discussion

### Key findings

When comparing the performance of the proposed method with the SPN and MIL baseline methods, the proposed method and the Ablation (Seeds) method significantly outperformed SPN and MIL in both Dice and HD95. The improved performance indicates that the SPN and MIL methods, while being effective in tasks with large training datasets, can worsen in tasks with limited available data such as brain tumor segmentation. MIL is frequently used for weakly supervised segmentation of histopathology images because of the need to interpret the large gigapixel resolution images in patches. We believe the significantly reduced spatial dimensions and resolutions of the MR images negatively impacted the performance of the MIL baseline. The MR images lacked the resolution required to extract patches with sufficient information that only occupied a small portion of its source image. As such, the MIL baseline was unable to effectively learn to segment the tumors.

PatchConvNet also suffered from the smaller dataset size. The PatchConvNet classifier was not able to generate effective undersegmented positive and negative seeds to guide the training of the superpixel generator and clustering models. This can be attributed to the smaller dataset size, which PatchConvNet was not designed for, and the use of attention-based maps for the seeds. With the smaller dataset size, PatchConvNet was unable to acquire an effective understanding of the tumors. As a result, the attention maps acquired from PatchConvNet did not consistently undersegment the cancerous and non-cancerous regions, which is a critical assumption when using the seeds. Using a VGG-16 classifier and generating the seeds using RISE resulted in localization seeds that tend to undersegment the cancerous and non-cancerous regions despite the limited available data.

CAM-S achieved the highest Dice and the lowest HD95 among the baseline methods, having produced seeds that outperformed the positive RISE seeds in both Dice and HD95. However, the proposed method trained with the RISE seeds outperformed the proposed method trained with the seeds from the CAM-S method. We attribute the difference in performance to the fact that the positive RISE seeds better undersegmented the tumors compared to the CAM-S seeds. The proposed method assumes that the positive seeds undersegment the tumors and the negative seeds undersegment the non-cancerous regions. The undersegmentation creates uncertain regions that add a margin of error for the seeds, which mitigates potential propagation errors originating from imperfect seeds.

SAM achieved similar performance to CAM-S and successfully segmented some images but failed to segment difficult cases as shown in Fig. 2. These results indicate that methods such as RISE can be used to automatically generate points which can be used as prompts for foundational segmentation models. However, the inability for SAM to segment multimodal images and the poor performance of SAM on smaller, less distinct tumors demonstrates the need for models developed and trained for specific medical tasks, especially in weakly-supervised contexts. It should be noted that SAM was the only method to have reasonable performance on incorrectly classified images, which is expected due to SAM being the only pretrained model among the evaluated models, and therefore being unaffected by the classifier’s performance.

The use of simultaneously generated superpixels is a key novelty of our work. When using traditional superpixel generation algorithms, the precision of the segmentations is dependent on the number of superpixels, as fewer superpixels can result in less refined boundaries. Training a deep learning model to generate superpixels simultaneously with a superpixel clustering model allows for the gradients of the loss functions that encourage accurate segmentations to propagate through the superpixel generation model. This allows the superpixel generation model to not just learn to generate superpixels, but also to generate a lower number of superpixels with refined boundaries around the tumors. Thus, simultaneous generation and clustering of superpixels using neural networks improves the segmentation performance when using superpixels for segmentation.

Figure 2 demonstrates how the proposed method can reduce the number of outputted superpixels despite using a predefined number of superpixels. In our test cohort, the models reduced the number of superpixels from a predefined limit of 64 to approximately 22 per image by outputting 64 superpixels but having the majority of superpixels have no associated pixels.

In Fig. 2, the superpixels do not perfectly contour the segmented regions because the segmentations are calculated using a weighted sum of the superpixel scores based on their association with each pixel. Thus, pixels whose most associated superpixel is not primarily a part of the segmented region can be segmented so long as it has a sufficiently high association score with the primarily segmented superpixel. As such, the segmentations cannot be generated simply by selecting superpixels outputted by the method, they need to be soft clustered using the superpixel association and weights. Despite the lower number of superpixels when using higher seed loss weights, the method is still able to segment smaller tumors. It can also be seen that superpixels outside the tumor regions do not align with brain subregions or local patterns. This indicates that the superpixels are tuned to segment specifically brain tumors. While Fig. 2 implies that only one superpixel is approximately required for each image, we argue that the clustering component has the benefit of allowing this method to be applied to tasks with multiple localized anomalies in each image.

### Limitations

A limitation of this method is its reliance on superpixels which are computed based on pixel intensity. While the superpixels provide valuable information that improves segmentations of brain tumors, the superpixels also provide constraints on the set of problems this method can be applied to. In particular, this method would be ineffective for segmenting non-focal ROIs.

In addition, the proposed method relies on the localization seeds to be trained effectively. Despite not requiring the localization seeds during inference, poor localization seeds during training can lead to poor segmentations during inference. In addition, no steps were taken to improve the RISE seeds. We consider the seed generation beyond the scope of this work but note that the lack of seed refinement could lead to propagation errors. The performance of the PatchConvNet baseline demonstrates the importance of seed accuracy. PatchConvNet was unable to output effective localization seeds for this specific task and using the seeds from PatchConvNet with our proposed method decreased the Dice coefficient from 0.691 to 0.134 on the test cohort. As such, effective localization seeds from an accurate classifier that undersegment the cancerous and non-cancerous regions are crucial for effective performance using the proposed method.

Another limitation is that this method cannot be trained end-to-end. While the method is a weakly supervised approach as it does not require any segmentation ground truths to train, it can also be interpreted as a fully supervised classification task followed by an unsupervised superpixel generation and clustering task. Without seeds generated from an accurate classifier, the downstream models will fail. Many clinical contexts have classifiers available that can be applied to this method. However, the proposed method cannot be applied to contexts without classifiers that require end-to-end training.

A shortcoming of this study is its use of 2D images rather than 3D images due to the GPU memory costs required to generate 3D superpixels using an FCN-based superpixel generation model. The method is not limited to 2D images and thus it is of interest to explore applications of this method in 3D contexts. Previous studies have demonstrated that 3D segmentation leads to superior performance compared to 2D segmentation, which suggests that this method could be improved further when applied to 3D images [45].

As is the case with other weakly supervised segmentation methods, the performance of our proposed method does not match the performance of fully supervised training. However, weakly supervised segmentation methods serve a different purpose than fully supervised segmentation methods. Weakly supervised segmentations are very effective at generating initial segmentations that can be revised by radiologists or for downstream semi-supervised training to reduce workload on medical datasets that lack manual annotations. In summary, despite the lower performance of our proposed method compared to fully supervised training, our proposed method is effective for generating initial segmentations when manually annotated training data is not available.

Integrating multiple stages and modules into the pipeline increases the complexity of the proposed method. In future work, the complexity and running time of the pipeline, and the effect of implementing parallel computation [46–48] should be investigated.

Regularization has been demonstrated to improve model robustness during training, with particular benefits in mitigating bias toward majority classes [49]. Such regularization could be applied to the classifier model to improve extracted localization seeds and further mitigate seed propagation errors. In addition, image preprocessing can be used to account for noise and artifacts that frequently occur in medical images [2, 3]. The MR images in the BraTS datasets used in this study were preprocessed and made available. When applying the proposed methodology to unprocessed medical images, preprocessing techniques such as stochastic resonance theory [2, 50–52] have the potential to improve performance and should be explored further in future work.

## Conclusions

We introduced a weakly supervised superpixel-based approach to segmentation that incorporates contextual information through simultaneous superpixel generation and clustering. Integrating superpixels with localization seeds provides information on the boundaries of the tumors, allowing for the segmentation of tumors only using image-level labels. We demonstrated that using undersegmented seeds as opposed to seeds that attempt to accurately contour tumors can mitigate propagation errors from suboptimal seeds and lead to improved performance. This work can be used to improve the development of future weakly supervised segmentation methods through the integration of deep superpixels.

## Data Availability

The BraTS 2020 dataset analysed during the current study is available through the following website, https://www.med.upenn.edu/cbica/brats2020/data.html. The BraTS 2023 dataset analysed during the current study is available through the following website, https://www.synapse.org/Synapse:syn51156910/wiki/627000.

## Abbreviations

2D: 2-dimensional
3D: 3-dimensional
BraTS: Brain tumor segmentation
CAM-S: CAM-Superpixels
Dice: Dice coefficient
FCN: Fully convolutional network
HD95: 95% Hausdorff distance
MIL: Multiple instance learning
ML: Machine learning
MLCN: Multi-level classification network
RISE: Randomized input sampling for explanation of black-box models
ROI: Regions of interest
SAM: Segment anything model
SLIC: Simple linear iterative clustering
SPN: Superpixel pooling network
T2-FLAIR: T2 fluid attenuated inversion recovery
U-Dice: Undersegmented Dice coefficient
